# Eco-environmental changes due to human activities in the Erhai Lake Basin from 1990 to 2020

**DOI:** 10.1038/s41598-024-59389-6

**Published:** 2024-04-15

**Authors:** Xiaojie Liu, Junyi Chen, Bo-Hui Tang, Liang He, Yunshan Xu, Chao Yang

**Affiliations:** 1 Faculty of Land Resource Engineering, Kunming University of Science and Technology, Kunming, 650093 China; 2 Surveying and Mapping Geo-Informatics Technology Research Center On Plateau Mountains of Yunnan Higher Education, Kunming, 650093 China; 3 School of Environmental Science, Nanjing Xiaozhuang University, Nanjing, 211171 China; 4 College of Landscape Architecture and Horticulture, Southwest Forestry University, Kunming, 650224 China; 5 MNR Key Laboratory for Geo-Environmental Monitoring of Great Bay Area & Guangdong Key Laboratory of Urban Informatics & Shenzhen Key Laboratory of Spatial Smart Sensing and Services, Shenzhen University, Shenzhen, 518060 China; 6 School of Architecture and Urban Planning, Shenzhen University, Shenzhen, 518060 China

**Keywords:** Eco-environmental quality, Ecological index, Erhai Lake Basin, Spatiotemporal evolution, Human activities, Environmental impact, Urban ecology, Ecology

## Abstract

Human activities have increased with urbanisation in the Erhai Lake Basin, considerably impacting its eco-environmental quality (EEQ). This study aims to reveal the evolution and driving forces of the EEQ using water benefit–based ecological index (WBEI) in response to human activities and policy variations in the Erhai Lake Basin from 1990 to 2020. Results show that (1) the EEQ exhibited a pattern of initial degradation, subsequent improvement, further degradation and a rebound from 1990 to 2020, and the areas with poor and fair EEQ levels mainly concentrated around the Erhai Lake Basin with a high level of urbanisation and relatively flat terrain; (2) the EEQ levels were not optimistic in 1990, 1995 and 2015, and areas with poor and fair EEQ levels accounted for 43.41%, 47.01% and 40.05% of the total area, respectively; and (3) an overall improvement in the EEQ was observed in 1995–2000, 2000–2005, 2005–2009 and 2015–2020, and the improvement was most significant in 1995–2000, covering an area of 823.95 km^2^ and accounting for 31.79% of the total area. Results also confirmed that the EEQ changes in the Erhai Lake Basin were primarily influenced by human activities and policy variations. Moreover, these results can provide a scientific basis for the formulation and planning of sustainable development policy in the Erhai Lake Basin.

## Introduction

Rapid urbanisation since the 1950s has caused significant disruptions in regional and global ecosystems, particularly in China^1–3^.The number of prefecture-level and county-level cities in China increased from 193 in 1978 to 673 in 2018, with the built-up urban area expanding by approximately 7.8 times^4^. In addition, the pattern of land resource allocation has been altered, and large ecological land areas, including grasslands and forests, have been converted into urban areas. Habitat problems such as the urban heat island effect, climate change and aquatic environment degradation have exacerbated, impacting the security of regional ecosystems^5–7^.

The ecological environmental status and ecosystem security in the region can be directly assessed by evaluating the ecological environmental quality (EEQ)^8,9^. Currently, regional EEQ evaluation primarily relies on field measurements and remote sensing techniques^10^. Field measurements can effectively and accurately assess the regional EEQ but often require extensive data collection as well as considerable time and manpower. In contrast, remote sensing techniques, with their advantages of rapid, real-time and large-scale monitoring, have been proven to be effective in rapidly capturing the spatiotemporal changes in regional ecological environments^11,12^.

Early studies on ecological environment monitoring based on remote sensing techniques often used only a single ecological indicator for assessing the regional EEQ^13,14^; however, it might not yield accurate results due to the complex interactions among ecosystems and the diversity of influencing factors. Ying et al.^15^ proposed a comprehensive evaluation method based on analytic hierarchy process (AHP) for determining EEQ, which integrated social economy and natural environmental factors. In 2015, the Ministry of Environmental Protection of the People’s Republic of China released and optimised the ecological index (EI), which was used to assess the EEQ in lake basins and cities^16,17^. However, most of the previously used methods have shortcomings, including cumbersome evaluation factors, difficulty in acquiring data and a high degree of subjectivity in indicator weights. Therefore, Xu^9^ selected four indicators, namely greenness, dryness, wetness and heat, based on the pressure-state-response (PSR) framework. These indicators were combined with PCA to create a remote sensing–based ecological index (RSEI) for evaluating the comprehensive ecological status of regions. Then, to address the issue of non-uniqueness in the eigenvector direction in PCA, Ning et al.^18^ proposed an RSEI that does not consider the direction of eigenvectors. Yang et al.^19^ adopted the PSR framework and incorporated five indicators, namely vegetation cover (VC), vegetation health index (VHI), normalised building and bare soil index (NDBSI), land surface humidity (LSM) and land surface temperature (LST), to construct a comprehensive ecological evaluation index (CEEI) for assessing the EEQ objectively.

Currently, the RSEI is widely used for EEQ evaluation in areas of varying scales, including nature reserves^20^, sea island cities^21^, and Northeast China^22^ owing to its portability. Furthermore, the RSEI was enhanced via effective modifications and its scalability was reported^23,24^. However, the EEQ of water cannot be effectively evaluated using the RSEI because water bodies are susceptible to producing outliers for various indicators; therefore, they must be masked before EEQ evaluation^25,26^. Water quality is one of the crucial factors affecting the regional ecological environment^27^; therefore, the impact of water bodies must be considered in evaluating the regional EEQ. To this end, Jiao et al.^26^ first developed the surface potential water abundance index (SPWI) to depict the spatial distribution of water-related ecological factors. Then, the entropy weight method was used to integrate the five indexes of SPWI, normalised difference latent heat index (NDLI), ratio vegetation index (RVI), normalised difference soil index (NDSI) and LST to produce a water benefit–based ecological index (WBEI). This method was used to conduct regional EEQ assessments, including water bodies. The effectiveness of WBEI was demonstrated via its comparative analysis with RSEI and EI. Unfortunately, Jiao et al.^26^ assessed the performance of WBEI holistically and did not provide a detailed analysis of the EEQ of water bodies. However, water is an integral part of natural ecosystems, and the EEQ of water bodies directly affects regional ecosystem security.

The Erhai Lake Basin is the second-largest plateau freshwater lake basin in China. It is the primary source of production and domestic water supply for Dali City and its surrounding villages^28^. In recent years, the rapid urbanisation in the Erhai Lake Basin, particularly in the lakeside area, has led to the significant expansion of urban areas and continuous degradation of the ecological environment. These developments have posed potential threats to the aquatic ecosystem and human health^29^. The EEQ in the Erhai Lake Basin was recently evaluated^30,31^, but the impact of water ecological factors was not considered therein. Erhai Lake and its surrounding rivers are integral components of the Erhai Lake Basin and must not be overlooked in its EEQ assessment. Water ecological factors must also be considered for EEQ assessment. In this study, the WBEI was used to determine the spatiotemporal characteristics of the EEQ in the Erhai Lake Basin from 1990 to 2020. In addition, the performance of WBEI in terrestrial and aquatic areas was assessed, and the EEQ in the Erhai Lake Basin was quantified by considering the impact of urbanisation on water bodies. Moreover, the spatiotemporal evolution characteristics and driving factors of EEQ were determined. These results can be used for sustainable regional development and improving the local ecological environment.

## Study area and data source

### Study area

**Figure 1:**
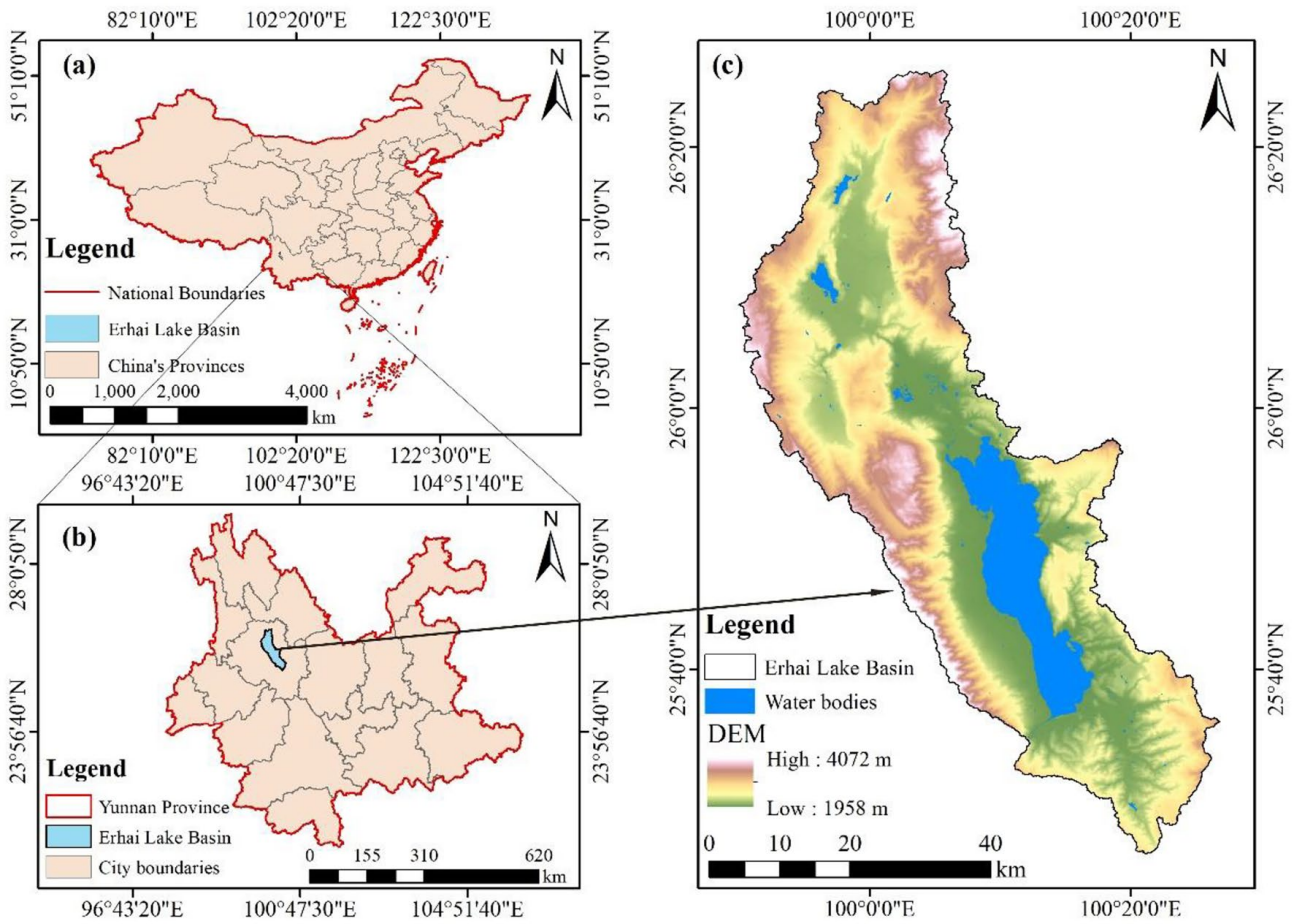
Location of the Erhai Lake Basin. (**a**) Study area in China, (**b**) study area in Yunnan Province and (**c**) elevation of the study area. The figure is created used ArcMap 10.7, https://www.arcgis.com.

The Erhai Lake Basin is located in the Dali Bai Autonomous Prefecture of Yunnan Province, Southwest China, spanning from latitude 25°36′N to 25°38′N and longitude 100°05′E to 100°17′E (Fig. 1). It is part of the Lancang–Mekong River system and experiences a low-latitude plateau subtropical monsoon climate. The temperature of the lake water remains between 10 °C and 20 °C throughout the year. As of 2020, the resident population of the river basin is approximately 9.9 million^32^.

### Data source and pre-processing

The Landsat 5/7/8 surface reflectance datasets and water surface data^33^ were obtained from Google Earth Engine (GEE). To mitigate the impact of cloud cover, images with the least cloud cover over the entire study area from March (dry season) 1990 to 2020 (Table 1) were selected, and cloud removal and quality screening were performed on these datasets. Then, the mean value of high-quality multi-year images taken during the same month was calculated and the Savitzky–Golay (SG) filter was applied^34^ to fill in any missing data. As a result, the remote sensing images of the Erhai Lake Basin from 1990 to 2020 with excellent quality and no cloud cover were obtained.

**Table 1:** Experimental data collection.

Date	Cloud (%)	GEE ID
1990.03.18	1.00	LANDSAT/LT05/C01/T1_SR
1995.03.16	0.00	LANDSAT/LT05/C01/T1_SR
2000.03.21	1.00	LANDSAT/LE07/C01/T1_SR
2005.03.11	5.00	LANDSAT/LT05/C01/T1_SR
2009.03.22	1.00	LANDSAT/LT05/C01/T1_SR
2015.03.23	0.06	LANDSAT/LC08/C01/T1_SR
2020.03.20	0.31	LANDSAT/LC08/C01/T1_SR
1990–2020	–	JRC/GSW1_3/YearlyHistory

## Methodology

**Figure 2:**
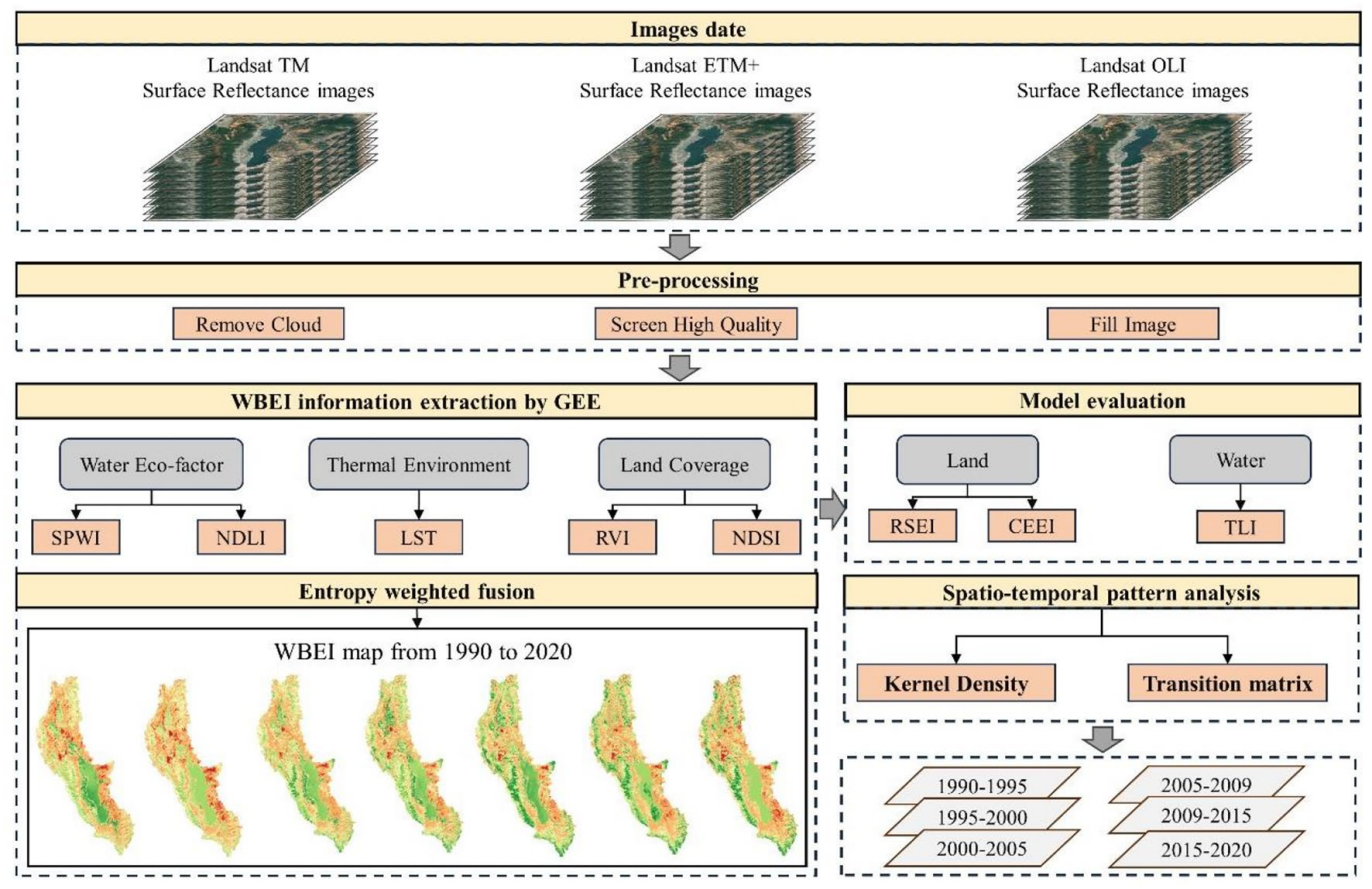
Flowchart of image processing and analysis.

A research framework was established to validate and assess the WBEI model performance, then monitor the EEQ evolution of the Erhai Lake Basin from 1990 to 2020. This framework encompasses several key components, including data pre-processing, WBEI calculation and assessment, EEQ classification and temporal and spatial monitoring of the EEQ (Fig. 2).

### WBEI construction method

The WBEI is a regional EEQ assessment index that integrates three key factors such as water ecological factors (SPWI and NDLI), thermal environment factors (LST) and land cover conditions (RVI and NDSI) using the entropy weight method.

#### SPWI

The SPWI is an indicator for the spatial distribution of surface water flow and provides insights into the influence of water on the ecological environment. It enhances the reliability of EEQ assessments in proximity to water bodies^26,35^ and is calculated as follows:

1 $$SPWI = \frac{{\mathop \rho \nolimits_{NIR} - \mathop \rho \nolimits_{SWIR2} + \mathop \rho \nolimits_{Blue} }}{{\mathop \rho \nolimits_{NIR} + \mathop \rho \nolimits_{SWIR2} + \mathop \rho \nolimits_{Blue} }},$$

where $\mathop \rho \nolimits_{Blue}$, $\mathop \rho \nolimits_{NIR}$ and $\mathop \rho \nolimits_{SWIR2}$ are the reflectance values of the blue, thermal infrared and second short-wave infrared bands of Landsat images, respectively.

#### NDLI

Urban air humidity considerably influences the characterisation of the internal climate of urban areas. Their impact on the urban ecosystem can be determined by investigating their correlation^36^. In this study, the NDLI was used to represent the urban air humidity^37^ ; it is calculated using Eq. (2):

2 $$NDLI = \frac{{\mathop \rho \nolimits_{Green} - \mathop \rho \nolimits_{Red} }}{{\mathop \rho \nolimits_{Green} + \mathop \rho \nolimits_{Red} + \mathop \rho \nolimits_{SWIR1} }},$$

where $\mathop \rho \nolimits_{Green}$,$\mathop \rho \nolimits_{Red}$ and $\mathop \rho \nolimits_{SWIR1}$ are the reflectance values of the green, red and first short-wave infrared bands, respectively.

#### LST

The urban heat island considerably influences the urban ecological environment, and its impact can be determined based on the LST^9^. Herein, the LST was retrieved using the emissivity modulation (EM) method^38,39^ as follows (Eqs. (3), (4), (5), (6)):

3 $$LST = \frac{{\mathop T\nolimits_{b} }}{{1 + \left( {\frac{{\lambda T_{b} }}{\rho }} \right)ln\varepsilon }}$$

4 $$\begin{array}{*{20}c} {\varepsilon = \left\{ {\begin{array}{*{20}c} {0.979 - 0.046 \times \mathop \rho \nolimits_{Red} } & {NDVI < 0.2} \\ {0.971(1 - P_{v} ) + 0.987P_{v} } & {0.2 \le NDVI \le 0.5} \\ {0.99} & {NDVI > 0.5} \\ \end{array} } \right.} \\ \end{array}$$

5 $$P_{v} = \left[ {\frac{{NDVI - NDVI_{min} }}{{NDVI_{max} - NDVI_{min} }}} \right]$$

6 $$NDVI = \frac{{\mathop \rho \nolimits_{NIR} - \mathop \rho \nolimits_{Red} }}{{\mathop \rho \nolimits_{NIR} + \mathop \rho \nolimits_{Red} }},$$

where $T_{b}$ and *λ* are the brightness temperature and central wavelength (μm) of the thermal infrared band, *ε* is the surface emissivity, *ρ* = 1.438 × 10^−2^ (mK), $P_{v}$ is the vegetation coverage, *NDVI*_*max*_ and *NDVI*_*min*_ are the maximum and minimum values of NDVI, respectively, and $\mathop \rho \nolimits_{NIR}$ and $\mathop \rho \nolimits_{Red}$ are the surface reflectance values in the near-infrared and thermal infrared bands, respectively.

#### RVI

The RVI effectively depicts vegetation coverage while mitigating discrepancies arising from various soil backgrounds and shadows^40^. Herein, the RVI was used to indicate the impact of regional human activities on the ecological models within regional boundaries; it is calculated using Eq. (7):

7 $$RVI = \frac{{\mathop \rho \nolimits_{NIR} }}{{\mathop \rho \nolimits_{Red} }},$$

where $\mathop \rho \nolimits_{Red}$ and $\mathop \rho \nolimits_{NIR}$ are the reflectance values of the red and near-infrared bands, respectively.

#### NDSI

In addition to vegetation cover, changes in impervious surface area can directly reflect the intensity of human activities on the ecological environment^41^. Herein, the NDSI, which is responsive to impervious surfaces, was used to determine the impact of human activities on the ecological environment^42^ and is calculated using Eq. (8):

8 $$NDSI = \frac{{\mathop \rho \nolimits_{SWIR1} - \mathop \rho \nolimits_{NIR} }}{{\mathop \rho \nolimits_{SWIR1} + \mathop \rho \nolimits_{NIR} }},$$

where $\mathop \rho \nolimits_{NIR}$ and $\mathop \rho \nolimits_{SWIR1}$ are the reflectivity values of the near-infrared and first short-wave thermal infrared bands, respectively.

### Corrected entropy weight coefficient method

Information entropy is a measurement index used to quantitatively describe the degree of information uncertainty and has been widely used in information fusion^43^. Herein, it was used to evaluate the information richness of each ecological indicator and the degree of information difference among the indicators. Each indicator was then assigned an appropriate weight^44^.

First, the weight of each indicator was calculated using the corrected entropy weight coefficient method^45^ as follows:

9 $$\begin{array}{*{20}c} {w_{j} = \frac{{1 - e_{j} }}{{m - \mathop \sum \nolimits_{i = 1}^{m} e_{j} }}\,\,\,\,\,\, j = 1,2,3, \ldots ,m} \\ \end{array}$$

10 $$\begin{array}{*{20}c} {e_{j} = \frac{1}{\ln \left( n \right)} \times \mathop \sum \nolimits_{i = 1}^{n} \left( {f_{ij} lnf_{ij} } \right)} \\ \end{array}$$

11 $$f_{ij} = \frac{{x_{ij} }}{{\mathop \sum \nolimits_{i = 1}^{n} x_{ij} }}$$

where *w*_*j*_ and *e*_*j*_ denote the weight and entropy for each ecological indicator *j*, respectively, *m* is the number of ecological indicators, *f*_*ij*_ is the proportion of pixel *i* in each ecological indicator *j*, *n* is the number of all pixels in each ecological indicator *j* and *x*_*ij*_ is the reflectivity of pixel *i* in each ecological indicator *j.*

As each indicator has a different unit and numerical range, their direct fusion will cause an imbalance of the indicator weight value^9^. Therefore, the values of the five indicators must be normalised to fall within [0,1] before fusion as follows:

12 $$\begin{array}{*{20}c} {N_{j} = \frac{{N_{i} - N_{min} }}{{N_{max} - N_{min} }}} \\ \end{array} ,$$

where *N*_*j*_ is the standardised value of *N*_*i*_, *N*_*max*_ and *N*_*min*_ represent the maximum and minimum values of each indicator, respectively.

The WBEI was then acquired by linear superposition and fusion of the weight value and the corresponding ecological indicator as follows:

13 $$\begin{array}{*{20}c} {WBEI = w_{1} \times N_{NDLI} + w_{2} \times N_{RVI} + w_{3} \times N_{SPWI} - w_{4} \times N_{LST} - w_{5} \times N_{NDSI} } \\ \end{array} ,$$

where NDLI, RVI and SPWI are positive indicators. LST and NDSI are negative indicators. $w_{1}$, $w_{2}$, $w_{3}$, $w_{4}$ and $w_{5}$ are the weight values of the NDLI, RVI, SPWI, LST and NDSI, respectively. $N_{NDLI}$, $N_{RVI}$, $N_{SPWI}$, $N_{LST}$ and $N_{NDSI}$ are the normalised values of the NDLI, RVI, SPWI, LST and NDSI, respectively.

### WBEI performance validation

Herein, the spatial coverage sampling method^46^ was used to evenly select 2000 points in the Erhai Lake Basin in 2020, and the WBEI performance for both water bodies and land areas was evaluated.

In the land area, the improved RSEI^18^ and CEEI^19^, which can quickly and accurately assess the improvement in regional terrestrial EEQ , were used to validate the performance of WBEI.

14 $$\begin{array}{*{20}c} {RSEI = PC1\left[ {f\left( {NDVI,Wet,NDSI,LST} \right)_{{\left| {V_{NDVI} } \right|, \, \left| {V_{Wet} } \right|, \, - \left| {V_{NDSI} } \right|, \, - \left| {V_{LST} } \right|}} } \right]} \\ \end{array}$$

15 $$CEEI = f\left( {VC,VHI,NDBSI,LSM,LST} \right)$$

where *PC1* is the first component of PCA, $f$ is the positive normalisation of the four indicators and $V_{NDVI}$, $V_{Wet}$, $V_{NDSI}$ and $V_{LST}$ are the eigenvectors of the NDVI, Wet^47^, NDSI and LST, respectively. VC is the vegetation coverage, VHI is the vegetative health index, NDSI is the normalised differential build-up and bare soil index^9^ and LSM is the land surface moisture. More detailed information about the RSEI and CEEI can be found in the study reported by Ning et al.^18^ and Yang et al.^19^.

In the water area, the trophic level index (TLI)^48^ was used to comprehensively validate the performance of WBEI in determining the EEQ.

16 $$C_{chl - a} = 67.519x^{2} + 16.995x - { 2}{\text{.0334}}$$

17 $$\begin{array}{*{20}c} {TLI = 10 \times \left[ {2.5 + 1.086 \times \log \left( {C_{chl - a} } \right)} \right]} \\ \end{array}$$

18 $$x = \frac{{\mathop \rho \nolimits_{NIR} }}{{\mathop \rho \nolimits_{Red} }},$$

where $x$ is the ratio of the near-infrared band to infrared band and $C_{chl - a}$ is the chlorophyll-a concentration in the water area.

### WBEI classification and EEQ change detection

Based on the principle that a higher value of EEQ (closer to 1) indicates a better ecological environment and by considering the mean and standard deviation of WBEI, the WBEI values were categorised into five ecological quality levels^49^ (Table 2). A transition matrix was used to describe the EEQ changes in the Erhai Lake Basin from 1990 to 2020^50^ (Table 3).

**Table 2:** Classification of the EEQ in the Erhai Lake Basin.

Types	Poor	Fair	Moderate	Good	Excellent
Dividing criteria	< $\overline{x}$−1.5 $s$	$\overline{x}$−1.5 $s$ ~ $\overline{x}$-0.5 $s$	$\overline{x}$-0.5 $s$ ~ $\overline{x}$ + 0.5 $s$	$\overline{x}$ + 0.5 $s$ ~ $\overline{x}$ + 1.5 $s$	> $\overline{x}$ + 1.5 $s$

$\overline{x}$ and $s$ represent the average value and standard deviation of the WBEI of EEQ from 1990 to 2020, respectively.

**Table 3:** Transition matrix of the EEQ in the Erhai Lake Basin.

T_1_-T_2_		T_1_				
		Poor	Fair	Moderate	Good	Excellent
T_2_	Poor	Unchanged	Degraded	Degraded	Degraded	Degraded
	Fair	Improved	Unchanged	Degraded	Degraded	Degraded
	Moderate	Improved	Improved	Unchanged	Degraded	Degraded
	Good	Improved	Improved	Improved	Unchanged	Degraded
	Excellent	Improved	Improved	Improved	Improved	Unchanged

T_1_ and T_2_ represent the beginning and ending years of each period, respectively.

### Kernel density estimation

Kernel density estimation (KDE) is a non-parametric method used to estimate the probability density function. It calculates the density distribution of points and line elements in space in their surrounding fields using a kernel function and reflects the distribution characteristics of spatial elements with the kernel density value of each grid in a continuous simulation image^51^.

19 $$\begin{array}{*{20}c} {f_{n} (x) = \frac{1}{{nh_{n} }}\sum\limits_{i = 1}^{n} {K\left( {\frac{{x - X_{i} }}{{h_{n} }}} \right)} } \\ \end{array} ,$$

where $f_{n} (x)$ is the estimated value of the probability density; $n$ is the number of observations; $h$ is the bandwidth, which is the extended width of $x$ in space and directly affects the precision of the kernel density result; $K( \cdot )$ is the kernel function and $x - X_{i}$ is the distance from $x$ to sample $X_{i}$.

## Results and discussion

### WBEI model performance

**Figure 3:**
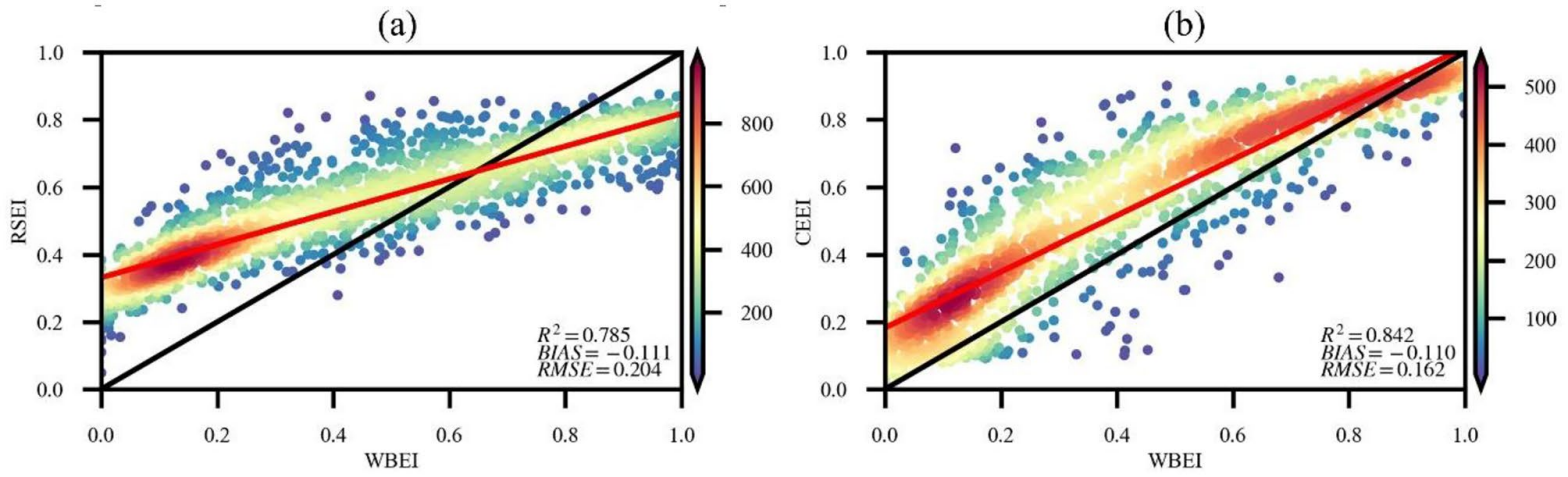
Correlation coefficients between WBEI and RSEI and CEEI. (**a**) WBEI with RSEI and (**b**) WBEI with CEEI.

**Figure 4:**
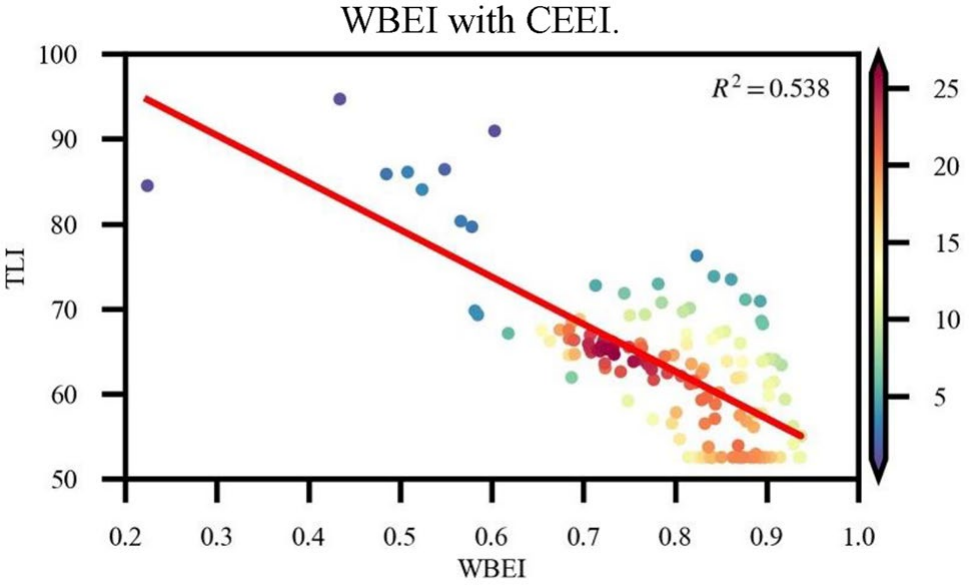
Correlation coefficient between WBEI and TLI.

To objectively assess the effectiveness of WBEI for EEQ assessment, distinct evaluation models were used for both land areas and water bodies. The correlation coefficients between the WBEI and RSEI as well as WBEI and CEEI were determined for land areas (Fig. 3), whereas that between the WBEI and TLI was determined for water bodies to assess the WBEI performance (Fig. 4). Thus, the WBEI had a strong correlation with the RSEI and CEEI in land areas, with R^2^ values of 0.785 and 0.842, respectively. It demonstrated a good correlation with TLI in water bodies (R = 0.733), with an R^2^ value of 0.538. These findings indicate that the WBEI can assess the quality of terrestrial ecological environments and water area ecological environments to a certain extent.

#### Validation of the SPWI

**Figure 5:**
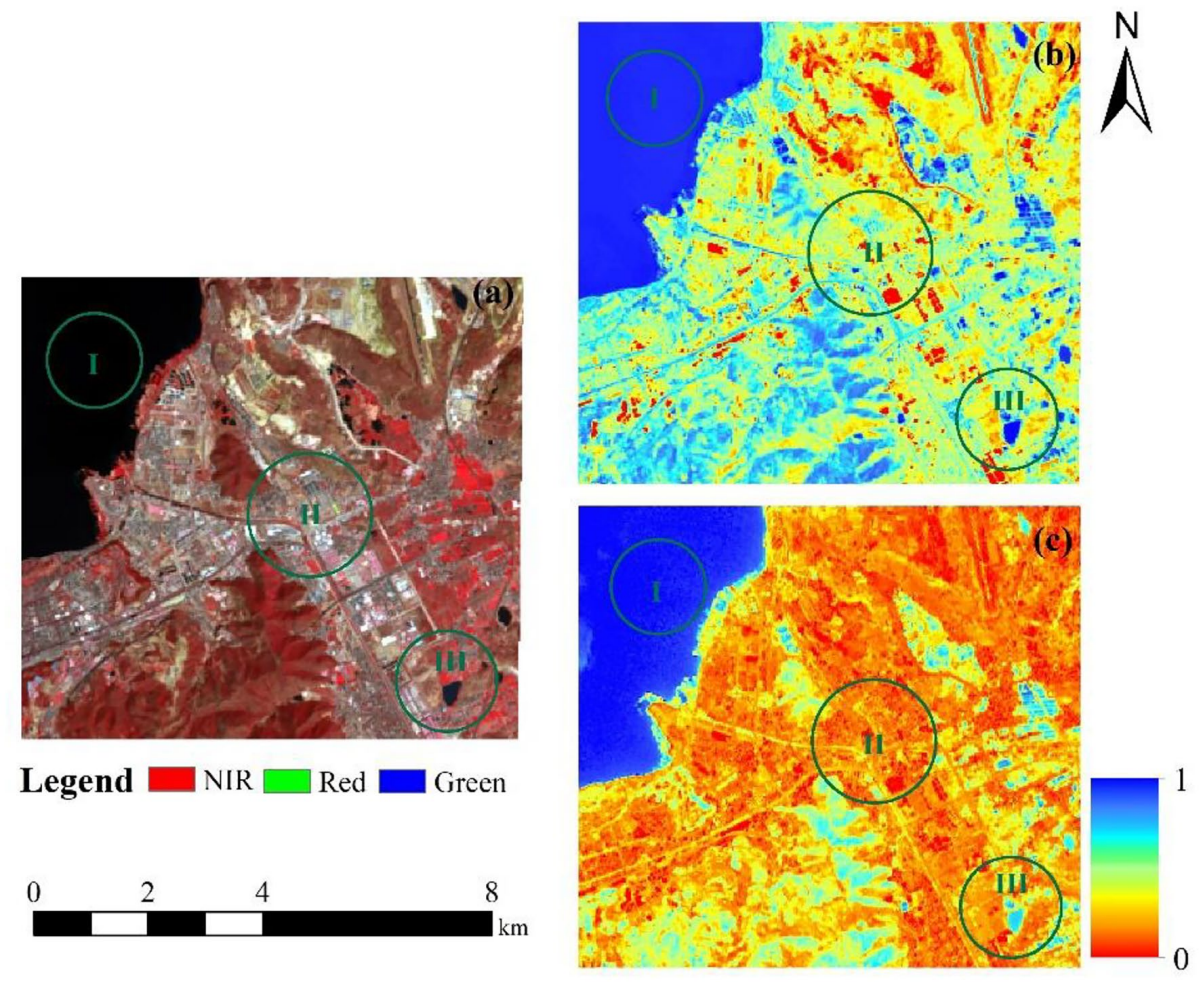
Spatial distribution of water obtained from (**a**) original false-color image, (**b**) Wet results, and (**c**) SPWI results.

To validate the ability of SPWI to characterize water spatial distribution, this study compared three different levels of water abundance in Erhai Lake, urban construction areas and ponds based on the Wet index^47^, which is widely used to evaluate regional EEQ. As shown in Fig. 5, in region (I) (Erhai Lake), the normalised SPWI results were consistent with the normalised Wet Index results, both indicating high water abundance. However, in region (II) (urban construction area), the water content abundance is extremely poor, whereas the Wet index results yielded high values, whereas SPWI yielded the opposite results. In addition, the water content abundance of region (III)(pond) should be much lower than that of region (I) (Erhai Lake), the values of the two regions were the same in the wet index results, while SPWI could effectively distinguish between the water abundance levels of the two regions. In summary, SPWI can better reflect the abundance of surface water resources in the Erhai Lake Basin.

### Spatiotemporal evolution of EEQ from 1990 to 2020

#### WBEI analysis

The average values of WBEI and the weights of each ecological factor from 1990 to 2020 were obtained using the GEE (Table 4). Based on the average WBEI value, the EEQ exhibited a pattern of initial degradation, subsequent improvement, further degradation and a rebound. Specifically, the changes in WBEI from 2009 to 2020 aligned with the changes in water resources monitored by the local water conservancy department^52^. This further confirmed the correlation between water factors and the regional ecological environment.

**Table 4:** Average values of the WBEI and the weight of each ecological factor from 1990 to 2020.

Year	Water Ecological Factors		Thermal Environment	Land Coverage		WBEI Mean
	SPWI	NDLI	LST	RVI	NDSI	
1990	0.22	0.30	0.10	0.28	0.10	0.37
1995	0.23	0.28	0.11	0.25	0.13	0.34
2000	0.23	0.24	0.12	0.29	0.12	0.40
2005	0.21	0.24	0.12	0.30	0.13	0.42
2009	0.22	0.22	0.18	0.27	0.11	0.45
2015	0.15	0.24	0.14	0.34	0.13	0.40
2020	0.17	0.28	0.14	0.26	0.15	0.41

Moreover, the weight of the water ecological indicator exhibited an initial improvement followed by degradation from 1990 to 2020, indicating significant fluctuations in the environmental water quality in the Erhai Lake Basin. The trend for LST weight from 1990 to 2020 was contrary to that for NDLI, in line with the explanation that LST decreases due to the cooling effect of evapotranspiration^37^. In terms of land cover, the combined weights of the RVI and NDSI exhibited a fluctuating upward trend, suggesting an increased responsiveness of land cover to the urban ecological environment in the Erhai Lake Basin over the past 30 years. This indicates that impervious surfaces encroached upon the natural landscape in the Erhai Lake Basin from 1990 to 2020.

#### Changes in the EEQ

**Figure 6:**
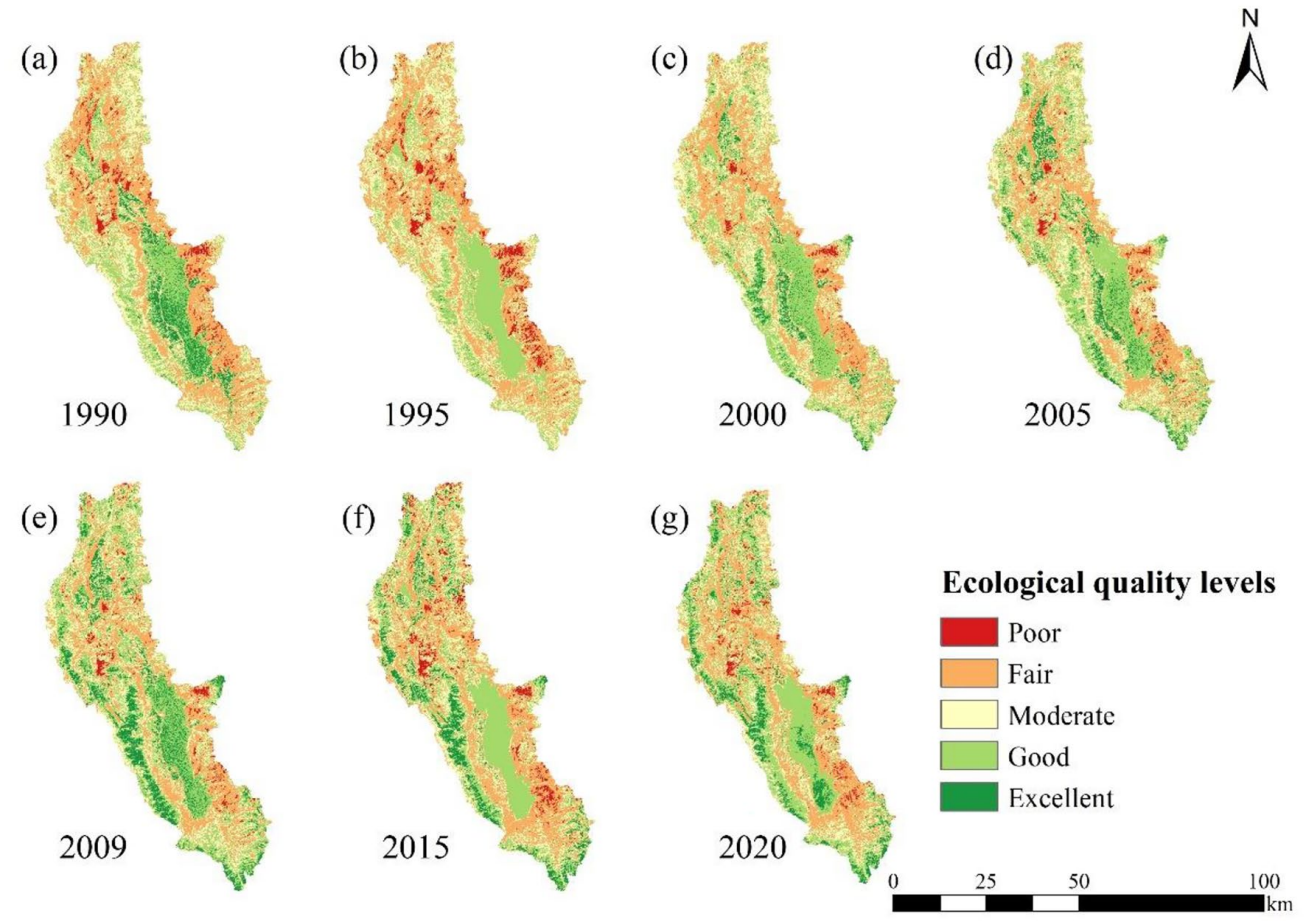
Spatiotemporal characteristics of the EEQ in the Erhai Lake Basin from 1990 to 2020.

**Figure 7:**
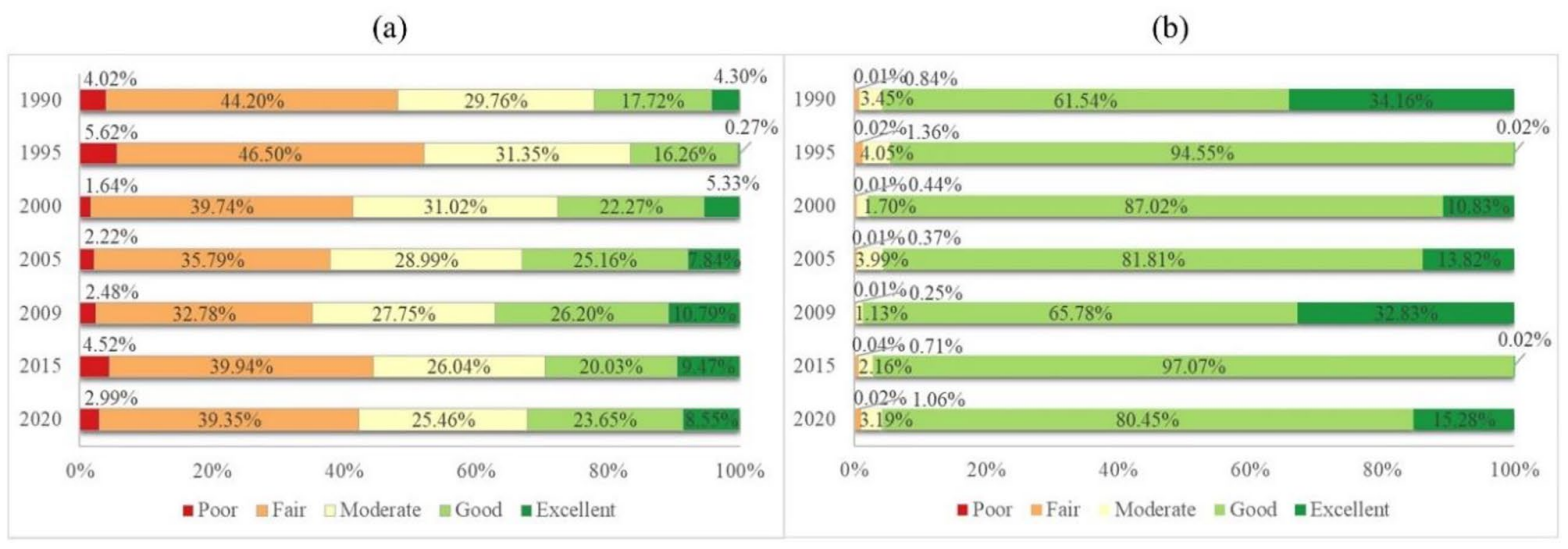
Changes in the EEQ levels of land areas and water bodies in the Erhai Lake Basin from 1990 to 2020. (**a**) Land and (**b**) water.

The spatiotemporal characteristics of the EEQ in the Erhai Lake Basin from 1990 to 2020 were derived from the WBEI classification results (Fig. 6), and the area and proportion of the EEQ classification were quantified (Table 5). The changes in the EEQ for each year were detailed from the perspectives of land areas and water bodies (Fig. 7). In general, from 1990 to 2020, the Erhai Lake Basin primarily contained regions with fair, moderate and good EEQ levels, accounting for more than 20%. Additionally, regions with poor and fair EEQ levels were primarily concentrated around Erhai Lake characterised by high urbanisation levels and relatively flat terrain.

**Table 5:** EEQ levels in the Erhai Lake Basin from 1990 to 2020.

Year	Poor		Fair		Moderate		Good		Excellent	
	Area (km^2^)	Percentage	Area (km^2^)	Percentage	Area (km^2^)	Percentage	Area (km^2^)	Percentage	Area (km^2^)	Percentage
1990	93.58	3.61	1031.71	39.80	702.23	27.09	574.67	22.17	190.07	7.33
1995	130.99	5.05	1086.78	41.92	740.59	28.57	627.51	24.21	6.40	0.25
2000	38.13	1.47	927.11	35.76	727.24	28.06	747.12	28.82	152.66	5.90
2005	51.76	2.00	835.03	32.21	685.94	26.46	800.58	30.88	218.95	8.45
2009	57.81	2.23	764.70	29.50	649.54	25.06	782.85	30.20	337.36	13.01
2015	105.57	4.07	932.78	35.98	612.42	23.63	720.64	27.80	220.86	8.52
2020	69.61	2.69	918.52	35.43	600.79	23.18	763.86	29.47	239.49	9.24

The Erhai Lake Basin exhibited lower EEQ levels in 1995 than in other years (Tables 4 and 5). A poor EEQ level was observed over an area of 130.99 km^2^, mainly concentrated along the eastern coast of Erhai Lake (Fig. 6). Specifically, the proportion land and water regions with poor EEQ levels accounted for 5.62% and 0.02% of the total land area and water body, respectively (Fig. 7). Meanwhile, areas with poor and good EEQ levels were the largest, reaching 1086.78 km^2^ (41.92%) and 740.50 km^2^ (28.57%), respectively (Table 5). The Erhai Lake Basin exhibited higher EEQ levels in 2009 than in other years. Excellent EEQ levels were observed across areas of 337.36 km^2^, accounting for 13.01% of the total area (Table 5), primarily concentrated in Erhai Lake and the Cangshan Mountain region characterised by lower levels of human activity (Fig. 6). Among them, the proportion of land and water with excellent EEQ levels accounted for 10.79% and 32.83% of the total land and water areas, respectively (Fig. 7). Previous studies have also confirmed our findings. Specifically, in the land regions from 1999 to 2019, lower EEQ values were predominantly clustered around Erhai Lake, which experienced intense human activity. Conversely, higher EEQ values were primarily concentrated in the western and southern parts of the study area, notably in the Cangshan region situated in the western segment of the Erhai Lake Basin^30,31^.

### Monitoring of dynamic changes in EEQ in the Erhai Lake Basin

**Figure 8:**
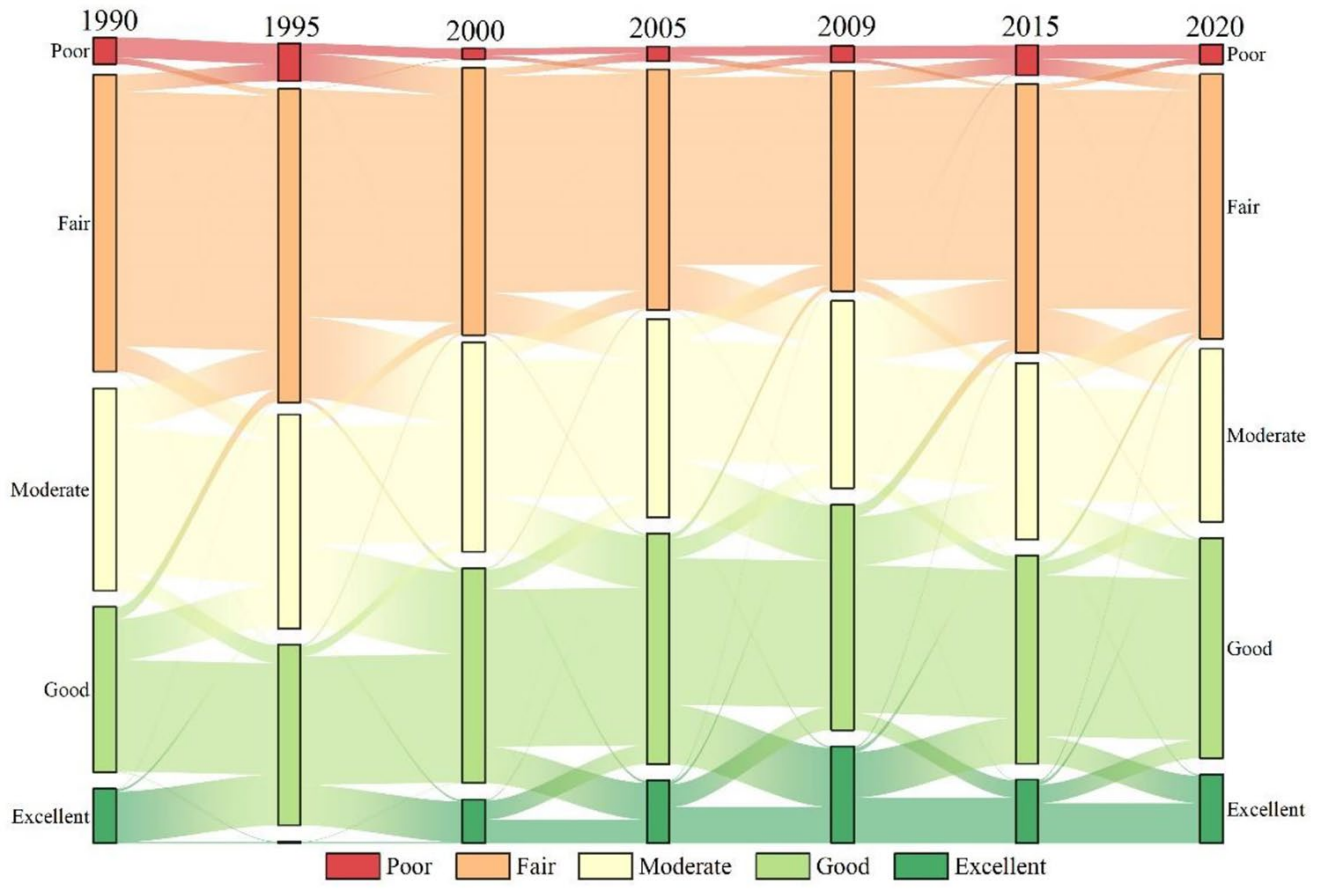
Sankey diagram of the EEQ changes in the Erhai Lake Basin from 1990 to 2020.

**Figure 9:**
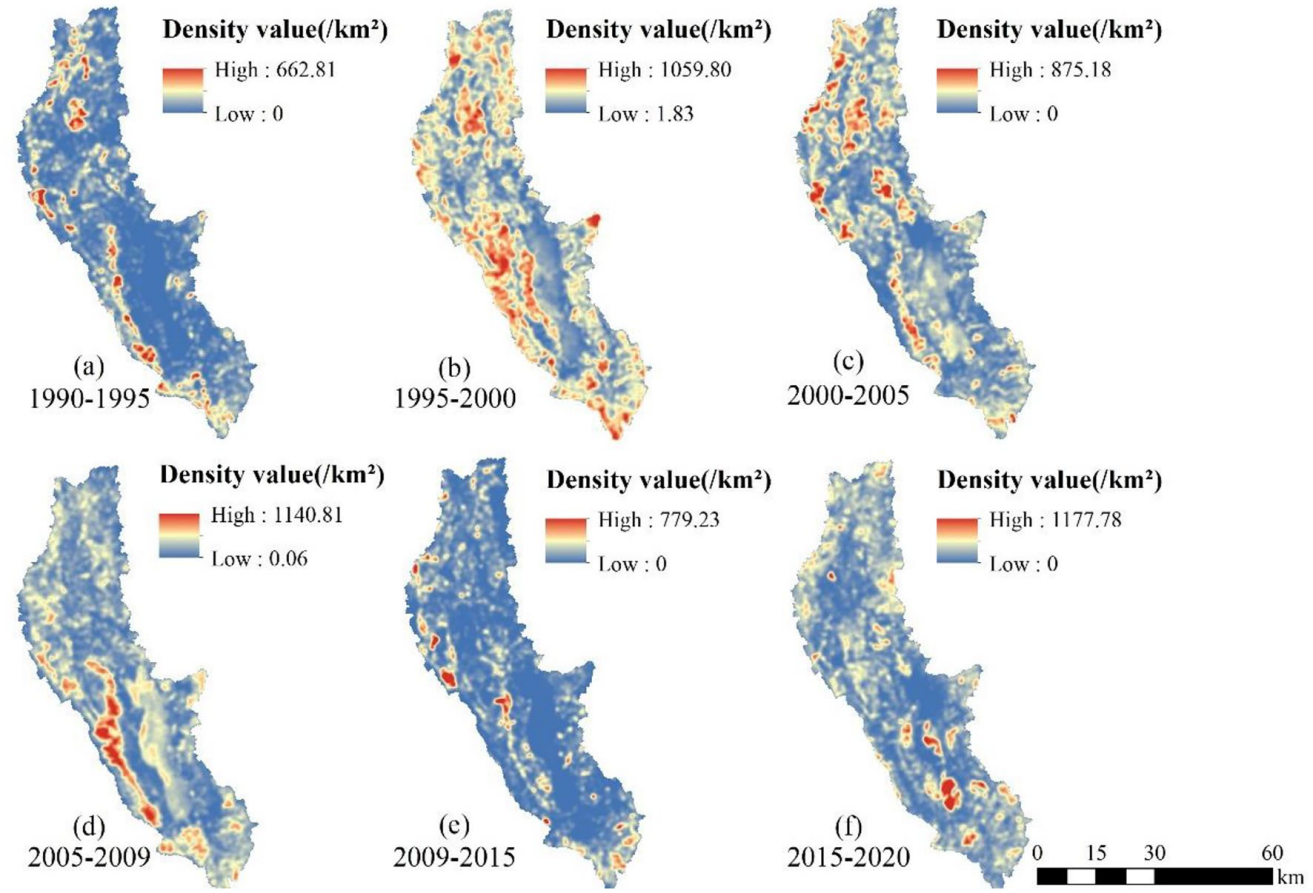
Kernel density distribution of EEQ improvement in the Erhai Lake Basin from 1990 to 2020.

**Figure 10:**
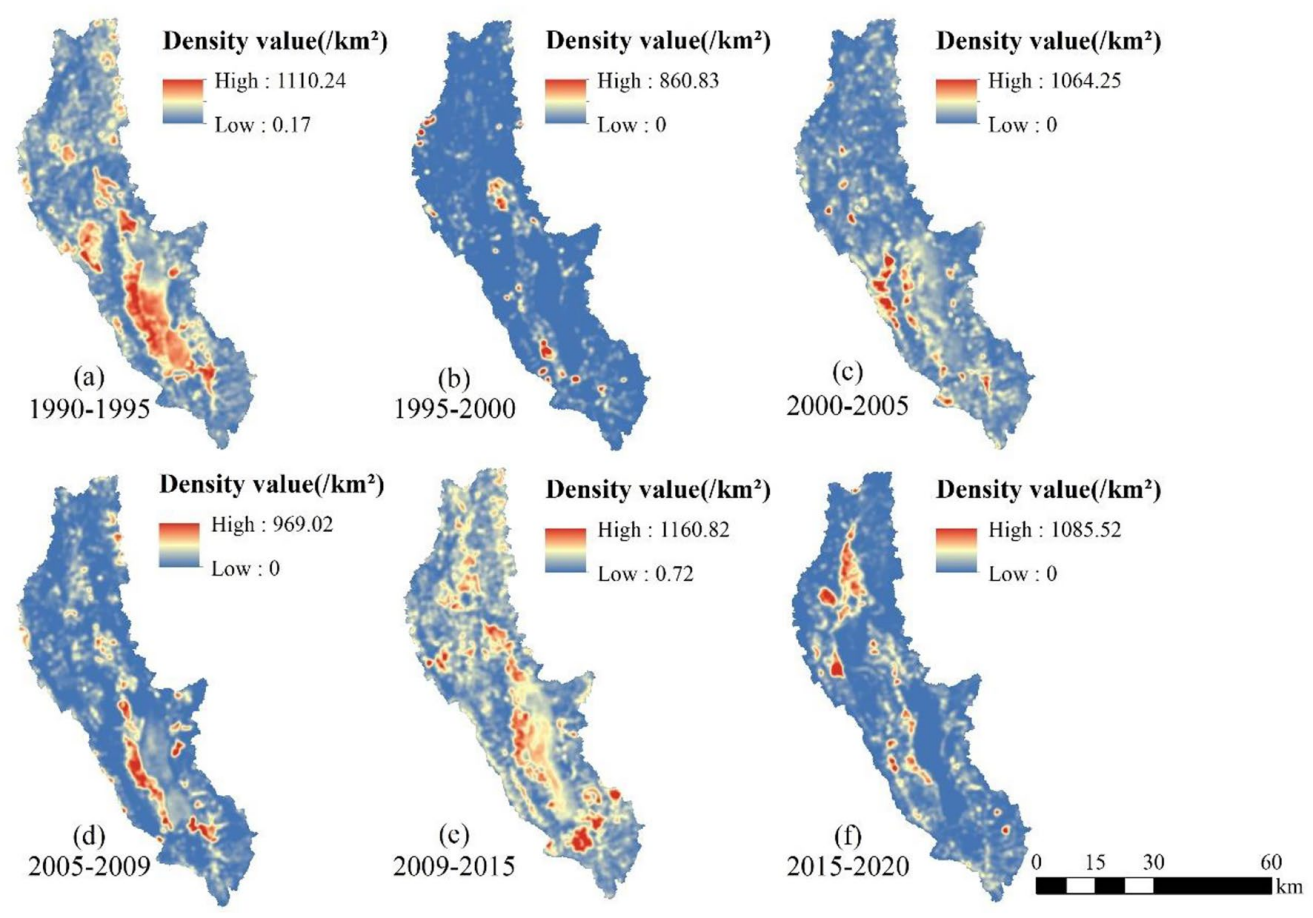
Kernel density distribution of EEQ deterioration in the Erhai Lake Basin from 1990 to 2020.

The EEQ changes in the Erhai Lake Basin from 1990 to 2020 were calculated using the transition matrix (Tables 6, 7 and Fig. 8), and a search radius of 1000 m was selected for KDE to visualise the spatial patterns of EEQ changes (Fig. 9 and 10). In general, the EEQ in the Erhai Lake Basin tended to deteriorate in 1990–1995 and 2009–2015 (Table 4). The areas (percentage) of deterioration were 567.72 km^2^ (21.90%) and 655.73 km^2^ (25.30%), respectively; these regions were mainly distributed around Erhai Lake (Fig. 10), particularly on its western coast. The EEQ tended to improve in 1995–2000, 2000–2005, 2005–2009 and 2009–2015 (Table 4), with the largest improvement in 1995–2000 across 823.95 km^2^, accounting for 31.79% of the total basin area. This area was concentrated in the southwest of the Erhai Lake Basin, particularly in the Cangshan Mountain region (Fig. 10). In addition, the improved area exceeded twice the deteriorated area in 2000–2005, 2005–2009 and 2015–2020 (Table 7).

**Table 6:** Transition matrix for the EEQ in the Erhai Lake Basin from 1990 to 2020.

1990–1995	1990					
1995	Area (km^2^)	Poor	Fair	Moderate	Good	Excellent
	Poor	71.24	59.22	0.40	0.13	0.00
	Fair	22.24	884.97	136.78	41.38	1.40
	Moderate	0.09	85.89	502.48	143.44	8.69
	Good	0.00	1.62	62.48	387.12	176.28
	Excellent	0.00	0.01	0.09	2.60	3.70

1995–2000	1995					
2000	Area (km^2^)	Poor	Fair	Moderate	Good	Excellent
	Poor	35.54	2.57	0.02	0.00	0.00
	Fair	94.75	788.49	37.43	6.40	0.04
	Moderate	0.68	281.07	411.50	33.71	0.28
	Good	0.03	14.45	284.67	446.25	1.73
	Excellent	0.00	0.20	6.97	141.14	4.35

2000–2005	2000					
2005	Area (km^2^)	Poor	Fair	Moderate	Good	Excellent
	Poor	23.81	27.76	0.16	0.01	0.02
	Fair	14.28	752.67	61.43	5.90	0.76
	Moderate	0.05	139.32	475.31	68.11	3.16
	Good	0.00	6.88	181.81	546.00	65.90
	Excellent	0.00	0.48	8.54	127.10	82.83

2005–2009	2005					
2009	Area (km^2^)	Poor	Fair	Moderate	Good	Excellent
	Poor	32.41	25.18	0.17	0.04	0.00
	Fair	19.10	653.57	70.90	18.10	3.02
	Moderate	0.19	153.49	417.30	67.81	10.78
	Good	0.05	2.74	192.59	508.47	79.01
	Excellent	0.00	0.05	4.98	206.18	126.15

2009–2015	2009					
2015	Area (km^2^)	Poor	Fair	Moderate	Good	Excellent
	Poor	45.40	56.45	2.79	0.88	0.05
	Fair	12.37	667.25	207.44	40.80	4.92
	Moderate	0.03	39.00	389.71	169.07	14.60
	Good	0.01	1.69	47.72	512.49	158.73
	Excellent	0.00	0.31	1.88	59.61	159.06

2015–2020	2015					
2020	Area (km^2^)	Poor	Fair	Moderate	Good	Excellent
	Poor	48.37	20.08	0.96	0.18	0.02
	Fair	56.37	759.00	80.65	17.33	5.16
	Moderate	0.73	147.10	391.34	50.15	11.46
	Good	0.09	5.97	135.02	558.52	64.26
	Excellent	0.00	0.63	4.44	94.45	139.96

**Table 7:** EEQ changes in the Erhai Lake Basin from 1990 to 2020.

Quality/year	Improved		Unchanged		Degraded	
	Area (km^2^)	Percentage	Area (km^2^)	Percentage	Area (km^2^)	Percentage
1990–1995	175.02	6.75	1849.52	71.35	567.72	21.90
1995–2000	823.95	31.79	1686.12	65.04	82.19	3.17
2000–2005	478.45	18.46	1880.62	72.54	233.19	9.00
2005–2009	579.37	22.35	1737.90	67.04	275.00	10.61
2009–2015	162.62	6.27	1773.91	68.43	655.73	25.30
2015–2020	444.81	17.16	1897.19	73.19	250.26	9.65

### Driving forces of the EEQ changes in the Erhai Lake Basin

The changes in EEQ in the Erhai Lake Basin were mainly influenced by a combination of anthropogenic and natural factors, with human activities and policy variations playing dominant roles^53^. This study conducted principal component analysis (PCA) on five indices (see Supplementary Table S1), revealing that the NDSI, which represents the intensity of human activities, exhibited the highest comprehensive contribution in the first principal component (PC1). This indicated that human activities and policy factors are the predominant drivers of EEQ changes in the Erhai Lake Basin, consistent with previous research findings^30,53^. Additionally, natural factors, such as climate, belong to the realm of macroscopic regulation and cannot be controlled, while anthropogenic factors, such as human activities, can be assessed and controlled through the mutual feedback between humans and the ecological environment^54^. Consequently, this study primarily analysed the influence of human activities and policy changes on the trends of EEQ changes.

Since the 1970s, the urbanisation rate in the Erhai Lake Basin has continuously increased. Moreover, human activities in the basin have remained consistently active, causing ecological environmental damage^55^. In 1990, 93.58 km^2^ (3.61%) and 1031.71 km^2^ (39.80%) of areas exhibited poor and fair EEQ levels, respectively (Table 5). Furthermore, the fishery has considerably increased in Dali City since 1993, and artificial fish feed is directly dispersed into Erhai Lake. This has deteriorated the water quality and increased the total nitrogen (TN) and total phosphorus (TP) concentrations^56^, leading to deteriorated EEQ.

The deteriorating ecological environment has affected the quality of life; thus, the importance of protecting the ecological environment to enhance the quality of life has been gradually recognised. The local government in China, with support from the United Nations Environment Programme (UNEP) and the United Nations Development Programme (UNDP), implemented ‘Investment Planning and Capacity Building for Sustainable Development of Erhai Lake Basin’ in 1995–1996. Since 2000, the Erhai Lake Basin has actively promoted tourism, leveraging its beautiful alpine lake scenery and distinctive ethnic culture. This has mitigated ecological degradation caused by agricultural and industrial development^57^, resulting in an overall improvement in the EEQ in the Erhai Lake Basin in 1990–2009. However, the absence of planning and delayed infrastructure development in the early rural areas around Erhai Lake has led to the rapid proliferation of hotels, inns and restaurants, resulting in disorderly and extensive development, as well as ecological degradation in non-coal mine^28,29^. Consequently, the EEQ in the Erhai Lake Basin seriously deteriorated from 2009 to 2015.

Fortunately, under the guidance of the Government of China to protect the ecological environment of the Erhai Lake Basin, the local government has proactively implemented a series of measures since the end of 2015. These measures include the establishment of sewage treatment plants, closure of mines, rectification of illegal construction works, and the formulation of a series of protection and treatment policies. On 30 May 2018, Dali City announced the ‘Three-line Delineation Plan for Ecological and Environmental Protection of Erhai Lake’ that defines the key management areas for water ecological protection. In May 2020, the government of the Dali Bai Autonomous Prefecture approved the implementation of the ‘Erhai Lake Protection and Governance Plan (2018–2035)’. This plan aims to provide directions for the protection and management of the Erhai Lake Basin, adopting a systematic and holistic approach to restore the lake basin and establish an ecological security barrier. As a result, due to the promotion and policy influence of local government departments, the EEQ in the Erhai Lake Basin considerably improved from 2015 to 2020.

### Development of ecological quality conservation measures

Water bodies, such as Erhai Lake and rivers, are crucial components of the Erhai Lake Basin. The water quality of these bodies is a key factor influencing the ecological environment within the basin. Therefore, considering the water bodies is helpful for a more comprehensive quantification of the EEQ within the Erhai Lake Basin. This, in turn, is of great significance for formulating effective measures to promote the sustainable development of the region. Based on the EEQ assessment results of the Erhai Lake Basin from 1990 to 2020 and the characteristics of the Erhai Lake Basin, this study proposes the following suggestions: (1) building a robust ecological security barrier, optimizing the urban green space pattern, and promoting sustainable development; (2) enhancing the effectiveness of the "Erhai Lake Protection and Governance Plan," including strengthening the construction of sewage treatment facilities, regulating the construction of hotels, restaurants, and other establishments, and enhancing publicity for Erhai Lake protection; (3) utilizing remote sensing and geographic information technology to establish a specialised monitoring system for the ecological environment management of the Erhai Lake Basin, achieving dynamic, effective, quantitative monitoring and evaluation, and providing rapid and timely feedback.

### Limitations and future works

Herein, the WBEI and EEQ in the Erhai Lake Basin were validated and determined, respectively, from 1990 to 2020; however, some limitations exist. Firstly, an empirical model was used to estimate the TLI. However, water bodies in plateau lakes exhibit highly complex water compositions and distinct regional characteristics^58^. In addition to chlorophyll-a, water transparency, TN and TP should be used to evaluate water quality^59^. These may be the reasons for the low correlation coefficient between the EEQ and TLI of water bodies. Secondly, the results of SG filtering were used to fill in the missing data. However, the details preserved after SG filtering may include abnormal information^60^, potentially introducing errors in the filled results.

For future studies, the combination of water transparency, chlorophyll-a, TN and TP should be considered to calculate TLI, further evaluate the performance of WBEI in assessing the EEQ of water bodies. WBEI could consider more water spectrum of different regions for improving the applicability of evaluating water EEQ. And more effective filtering methods such as weighted Whittaker smoother^60^ could be considered.

## Conclusions

In this study, the performance of WBEI in the EEQ assessment in land areas and water bodies was evaluated. Then, the spatiotemporal evolution characteristics of the EEQ from 1990 to 2020 in the Erhai Lake Basin were quantitatively analysed using WBEI, and the driving factors of the EEQ evolution were revealed. The spatiotemporal evolution characteristics of the EEQ exhibited a trend of initial degradation, subsequent improvement, further degradation and a rebound. The regions with poor and fair EEQ levels were mainly concentrated around Erhai Lake, which has a high urbanisation level and relatively flat terrain. Among them, the EEQ levels in 1990, 1995 and 2015 were not optimistic, and the regions with unsatisfactory EEQ (poor and fair) levels accounted for 43.41%, 47.01% and 40.05% of the total basin area, respectively. The EEQ level improved in 1995–2000, 2000–2005, 2005–2009 and 2015–2020. The improved area was the largest from 1995 to 2000, covering 823.95 km^2^ and accounting for 31.79% of the total basin area. By analysing the data on the EEQ in the Erhai Lake Basin from 1990 to 2020 and the planning documents of the local government on the management of the region, we confirmed that the EEQ was primarily affected by human activities and policy variations. The findings of this study can serve as a scientific basis for formulating sustainable development policies and the planning and management of the Erhai Lake Basin.

### Supplementary Information

Supplementary Table S1.

## Data Availability

All data, models, or codes generated or used during this study are available from the corresponding author upon reasonable request.
