# Supplementary material for: Eco-environmental changes due to human activities in the Erhai Lake Basin from 1990 to 2020
Source: Sci Rep. 2024 Apr 15;14:8646. doi: 10.1038/s41598-024-59389-6 (PMC11018612; doi:10.1038/s41598-024-59389-6)
Supplement: Supplementary file 1 — Supplementary Table S1. [file 41598_2024_59389_MOESM1_ESM.docx]

Supplementary Table S1. The first principal component results of five factors from 1990 to 2020.

| Year | PC1 | | | | | | |
| --- | --- | --- | --- | --- | --- | --- | --- |
|  | SPWI | NDLI | RVI | LST | NDSI | Eigenvalue | Percent eigenvalue  (%) |
| 1990 | -0.597 | -0.399 | 0.343 | -0.160 | 0.584 | 0.194 | 66.290 |
| 1995 | -0.568 | -0.429 | 0.342 | -0.133 | 0.599 | 0.226 | 70.030 |
| 2000 | -0.570 | -0.413 | 0.409 | -0.182 | 0.551 | 0.221 | 70.120 |
| 2005 | -0.569 | -0.402 | 0.356 | -0.235 | 0.577 | 0.201 | 67.070 |
| 2009 | -0.560 | -0.426 | 0.441 | -0.221 | 0.511 | 0.235 | 70.870 |
| 2015 | -0.496 | -0.440 | 0.407 | -0.310 | 0.546 | 0.291 | 73.520 |
| 2020 | -0.534 | -0.324 | 0.433 | -0.336 | 0.556 | 0.253 | 70.160 |
| Mean | -0.556 | -0.405 | 0.390 | -0.225 | 0.561 | 0.232 | 69.723 |
